# Preserving coronal knee alignment of the knee (CPAK) in unicompartmental knee arthroplasty correlates with superior patient-reported outcomes

**DOI:** 10.1186/s43019-023-00204-3

**Published:** 2024-01-02

**Authors:** Sung Eun Kim, Kuk-Ro Yun, Jae Min Lee, Myung Chul Lee, Hyuk-Soo Han

**Affiliations:** 1 Department of Orthopaedic Surgery, Seoul National University College of Medicine, 101 Daehak-Ro, Jongno-Gu, Seoul, 110-744 Republic of Korea; 2 Department of Orthopaedic Surgery, Seoul National University Hospital, Seoul, South Korea

**Keywords:** Unicompartmental knee arthroplasty, Coronal plane alignment, Patient-reported outcome measures

## Abstract

**Background:**

The optimal alignment target for unicompartmental knee arthroplasty (UKA) remains controversial, and literature suggests that its impact on patient-reported outcome measures (PROMs) varies. The purpose of this study was to identify the relationship between changes in the coronal plane alignment of the knee (CPAK) and PROMs in patients who underwent UKA.

**Methods:**

A retrospective analysis of 164 patients who underwent UKA was conducted. The types of CPAK types categorized into unchanged, minor (shift to an adjacent CPAK type, e.g., type I to II or type I to IV), and major changes (transitioning to a nearby diagonal CPAK type or two types across, such as type I to V or type I to III). PROMs were assessed preoperatively and 1 year postoperatively using the Hospital for Special Surgery (HSS) scores, Knee Society (KS) scores, Western Ontario and McMaster Universities Osteoarthritis Index (WOMAC), and Forgotten Joint Scores (FJS). Comparison was performed between patients who experienced and who did not experience any changes in the CPAK.

**Results:**

Patients with preserved native CPAK alignment demonstrated significantly superior 1 year postoperative outcomes, with higher HSS, KS knee, and WOMAC pain scores (*p* = 0.042, *p* = 0.009, and *p* = 0.048, respectively). Meanwhile, the degree of change in CPAK did not significantly influence the PROMs, and patients who experienced minor and major changes in the CPAK showed comparable outcomes.

**Conclusion:**

Preserving the native CPAK in UKA procedures is important for achieving favorable clinical outcomes at 1 year postoperative. The extent of change in the CPAK type exerted a limited impact on PROMs, thus emphasizing the importance of change in alignment itself.

## Introduction

Unicompartmental knee arthroplasty (UKA) is an effective surgical treatment for medial compartment osteoarthritis, and it offers advantages such as reduced surgical exposure, preservation of cartilage in unaffected knee compartments, retention of the cruciate ligaments, and biomechanics similar to the natural knee [1–5]. It is widely accepted that alignment affects the survivorship of UKA, but there is no consensus regarding the optimal alignment strategies for UKA [6–10].

Despite the endeavor to preserve native knee kinematics, UKA introduces alignment alterations through restoration of the joint space reduced by cartilage loss [11]. Conventional UKA adopts a mechanical alignment concept, aiming to achieve a neutral mechanical axis [12, 13]. This approach is based on the observation that 70% of the weight-bearing forces exert pressure on the medial compartment, and this load increases as varus alignment increases [14]. Consequently, undercorrection of varus alignment in medial UKA may result in early wear and failure of the polyethylene insert [15]. Meanwhile, recent studies have preferred a kinematic or pre-arthritic alignment strategy, involving resection of the femur and tibia to resemble their pre-arthritic state, with an aim to restore the joint to its state before cartilage degeneration [16, 17]. The normal alignment of the knee is naturally in slight varus [18]. In case of patients with constitutional varus alignment, uniform correction to neutral alignment may not be desirable [17].

The coronal plane alignment of the knee (CPAK) classification method determines a patient’s constitutional alignment by measuring the medial proximal tibial angle (MPTA) and lateral distal femoral angle (LDFA) [19]. By determining the difference between the MPTA and LDFA and then summing them up, the resultant arithmetic hip–knee–ankle angle (aHKA) and joint line obliquity (JLO) can be classified into nine types. These categories include a range of aHKAs (varus, neutral, and valgus) and JLO (apex distal, neutral, and apex proximal) [19]. The principles of mechanical or kinematic alignment in UKA can potentially integrate with the CPAK, as UKA may change the type of CPAK, thus potentially influencing patient outcomes [20]. To the best of our knowledge, none of the studies have yet explored the clinical outcomes of patients who underwent UKA in relation to a shift in the CPAK classification.

Therefore, the purpose of this study was to evaluate the patient-reported outcome measures (PROMs) in patients who underwent UKA, by comparing those with changes in the CPAK phenotype with those who did not experience any changes in the CPAK phenotype. In addition, a subgroup analysis categorized patients into those with a minor shift to an adjacent CPAK type and those with major extensive transitions in CPAK type. The aim of this subgroup analysis was to identify potential differences in PROMs based on the extent of changes in the CPAK. We hypothesized that patients with any CPAK change would exhibit poorer outcomes than those without, and that major changes would lead to worse outcomes than minor ones.

## Materials and methods

### Data collection

This study was approved by the institutional review board (IRB) of the authors’ institute (IRB no. 2005-180-1126). The requirement of obtaining informed consent was waived due to the retrospective nature of the study. We conducted a retrospective review of patients who underwent medial UKA between January 2005 and December 2020, with a minimum 1 year postoperative follow-up. The selection criteria for UKA included patients with medial knee pain due to medial compartment osteoarthritis or osteonecrosis (over Kellgren and Lawrence grade 2), a coronal plane deformity of less than 15 degrees, a flexion contracture of less than 15 degrees, intact cartilage in the lateral compartment and patellofemoral joint as confirmed by magnetic resonance imaging (MRI), and an intact anterior cruciate ligament, also verified by MRI.

**Fig. 1:**
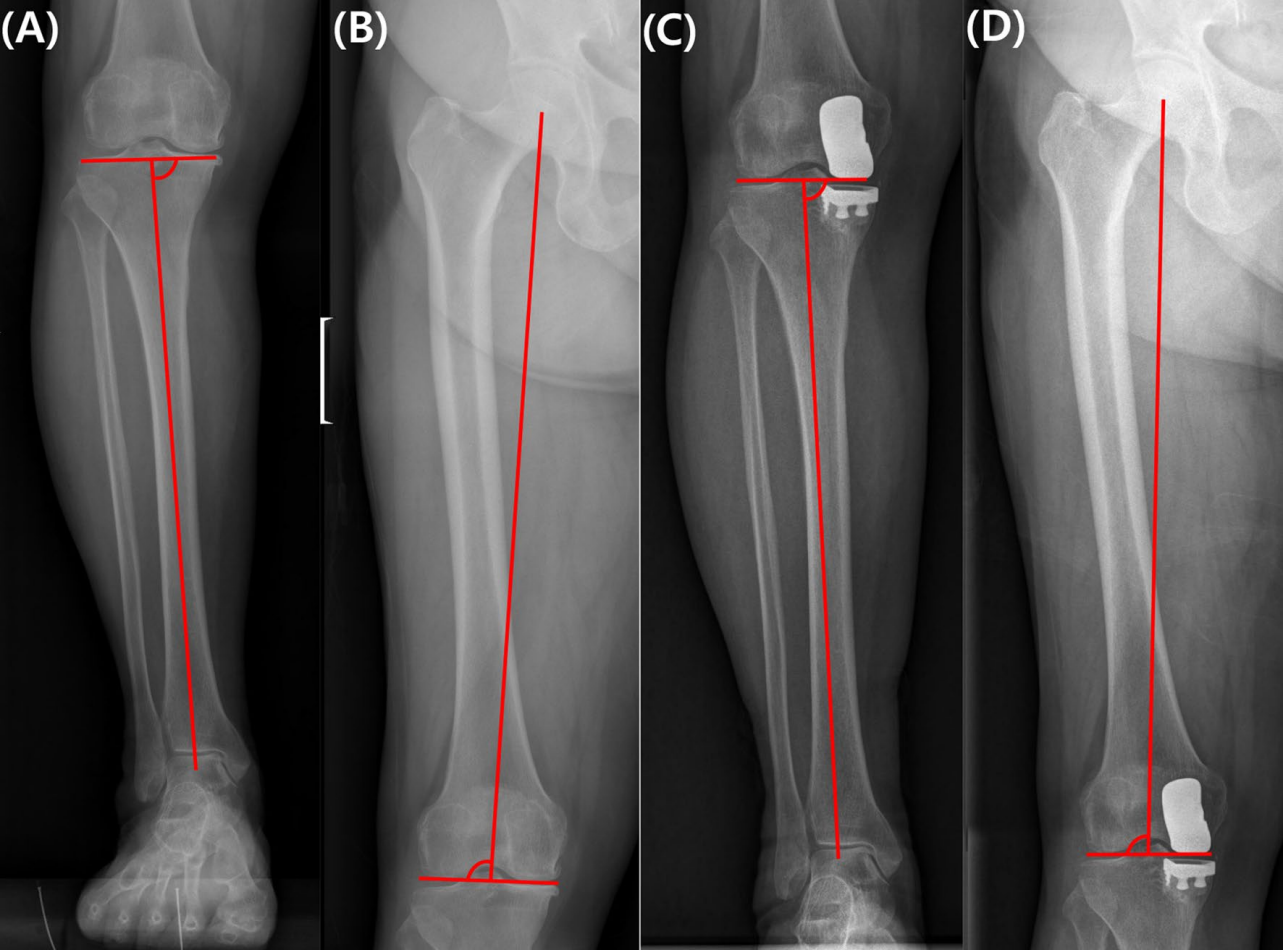
Radiologic parameter measurements: **A** Preoperative MPTA, **B** preoperative LDFA, **C** postoperative MPTA, and **D** Postoperative LDFA. In **C** and **D** the joint lines were measured using a line extending from the distal end of the femoral component to the center of the lateral joint space. MPTA, medial proximal proximal tibial angle; LDFA, lateral distal femoral angle

Patient demographic data, including age, sex, and body mass index (BMI), were collected. Preoperative and postoperative 1 year MPTA and LDFA values from long-leg radiographs were measured. The MPTA was defined as the angle between the proximal joint line and the tibial mechanical axis, while the LDFA was defined as the lateral angle between the distal joint line and the femoral mechanical axis. Postoperative measurements were adjusted due to radiopaque femoral and tibial implants, along with the radiolucent polyethylene insert. The postoperative MPTA was defined as the angle between the line from the distal end of the femoral component to the center of the lateral joint space and the tibial mechanical axis. The postoperative LDFA was defined as the lateral angle between the line from the distal end of the femoral component to the center of the lateral joint space and the femoral mechanical axis. (Fig. 1). As both the medial tibial and femoral cartilages, along with the subchondral bones, are resected during UKA, we used the distal end of the femoral component in the medial compartment as a reference point for both MPTA and LDFA. This point contacts the polyethylene insert, serving as a reliable reference. The CPAK type was categorized based on the classification by MacDessi et al., with types I through IX representing the nine alignment phenotypes [19]. The PROMs of patients who retained their CPAK classification after surgery and who experienced changes in the CPAK classification after surgery were compared. Additionally, a subgroup analysis was performed by categorizing patients who experienced a shift in the CPAK classification: those with minor changes (shift to an adjacent CPAK type, e.g., type I to II or type I to IV) and those with major changes (transitioning to a nearby diagonal CPAK type or two types across, such as type I to V or type I to III).

PROMs, including the Hospital for Special Surgery (HSS) scores [21], Knee Society (KS) scores (knee scores and function scores) [22], the Western Ontario and McMaster Universities Arthritis Index (WOMAC) [23], and the Forgotten Joint Scores (FJS) [24], were documented both preoperatively and at 1 year postoperatively. Complications that necessitated revision surgery were also recorded until the final follow-up of each patient.

### Surgical protocol

All UKAs were performed by two experienced surgeons at a high-volume tertiary hospital. A medial mini-parapatellar arthrotomy was performed (10 cm), followed by osteophyte resection. Medial release was performed only for the deep medial collateral ligament. Tibial resection was carried out using extramedullary guides to achieve matched mediolateral declination and anteroposterior tibial slope. The target for tibial resection was set at a thickness of 4 mm to achieve a minimum gap of 8 mm. Mediolateral inclination was adjusted by manipulating the distal end position of the extramedullary guide. In instances where the preoperative MPTA exhibited valgus alignment, the resection was aligned with the native mediolateral declination, as previously mentioned. Notably, the maximum MPTA was 92 degrees in this study, and instances of valgus MPTA were rare. Distinct techniques for distal femoral resection were employed based on the implant used during surgery. For one approach, an intramedullary guide was used, and the distal femoral cut was determined by the valgus angle established on the basis of preoperative standing long-leg radiographs (MIS Miller/Galante, Zimmer, Warsaw, USA). The other technique utilized a spacer block to achieve well-balanced flexion and extension gaps (Sigma High Performance Partial Knee, DePuy Synthes, Warsaw, USA). All components were fixed with cement. Patients were encouraged to walk and perform range of motion exercises as tolerated on the day of surgery.

### Statistical analysis

Statistical analysis was performed using Python 3.11.4. Categorical variables were evaluated using the chi-square method, while continuous variables were analyzed using the independent *t*-test and the one-way analysis of variance (ANOVA). Post hoc analysis was conducted using Tukey’s Honestly Significant Difference (HSD) method. Two blinded independent observers conducted radiographic measurements, and the intraclass correlation coefficient (ICC) was subsequently calculated to assess the level of agreement between measurements. A *p*-value below 0.05 was considered statistically significant.

## Results

The demographic characteristics, preoperative data, and radiologic characteristics of patients included in this study are presented in Tables 1 and 2. During the study period, 173 patients were initially assessed for eligibility, and 9 patients were excluded due to missing follow-up records. Finally, 164 patients were included in the analysis. The ICC values for radiologic parameters were 0.943, 0.943, 0.779, and 0.888 for preoperative MPTA, preoperative LDFA, postoperative MPTA, and postoperative LDFA, respectively, and they indicated good agreement between measurements.

**Table 1:** Demographic characteristics and preoperative data of patients

*N* = 164	Mean ± SD	Range
Sex (male %)	22 (13.4%)	
Age (years)	66.5 ± 7.6	37.6–85.5
Weight (kg)	63.8 ± 10.0	38.4–109.4
Height (cm)	155.7 ± 6.8	140.0–179.3
Body mass index (kg/m^2^)	26.3 ± 3.3	18.1–39.8
Follow-up duration (months)	19.8 ± 23.0	12.0–151.8
Preoperative knee ROM	128.5 ± 13.1	90–150
Postoperative knee ROM	130.5 ± 11.5	100–150
Preoperative HSS score	62.5 ± 15.2	11–89
Preoperative KS knee score	54.1 ± 16.8	15–90
Preoperative KS function score	38.0 ± 16.3	3–86
Preoperative WOMAC pain	10.7 ± 4.3	2–27
Preoperative WOMAC stiffness	4.0 ± 2.0	0–10
Preoperative WOMAC physical function	33.2 ± 7.9	4–50
Preoperative WOMAC total	47.9 ± 10.5	14–72
Preoperative FJS	11.1 ± 10.4	2.1–31.3

SD, standard deviation; ROM, range of motion; HSS, the Hospital for Special Surgery; KS, the Knee Society; WOMAC, the Western Ontario and McMaster Universities Arthritis Index; FJS, Forgotten Joint Score

**Table 2:** Preoperative and postoperative radiologic measurements of patients

*N* = 164	Preoperative		Postoperative		*p*-Value
	Mean ± SD	Range	Mean ± SD	Range	
MPTA	87.2 ± 1.9	82.1–92.0	86.8 ± 1.8	82.8–92.0	0.018*
LDFA	88.7 ± 1.8	84.0–93.7	89.0 ± 2.2	83.5–95.0	0.029*
PTS	10.7 ± 3.1	0.3–21.8	8.1 ± 2.9	0–13.2	< 0.001*
JLO	175.8 ± 2.9	167.4–185.7	175.8 ± 3.1	168.4–183.3	0.993
HKA	−6.0 ± 3.2	−15.0 to 5.9	-2.1 ± 3.0	−11.0 to 5.3	< 0.001*
aHKA	−1.5 ± 2.4	−8.6 to 5.8	−2.1 ± 2.7	−11.2 to 3.5	0.001*

Negative values indicate varus angulation.

SD, standard deviation; MPTA, medial proximal tibial angle; LDFA, lateral distal femoral angle; PTS, posterior tibial slope; JLO, joint line obliquity; HKA, hip–knee–ankle angle; aHKA, arithmetic hip–knee–ankle angle.

*Statistically significant at p < 0.05

**Fig. 2:**
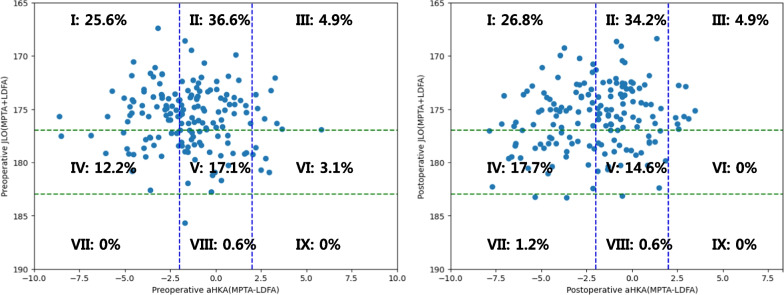
Preoperative and postoperative distribution of coronal plane alignment of the knee (CPAK) types

**Fig. 3:**
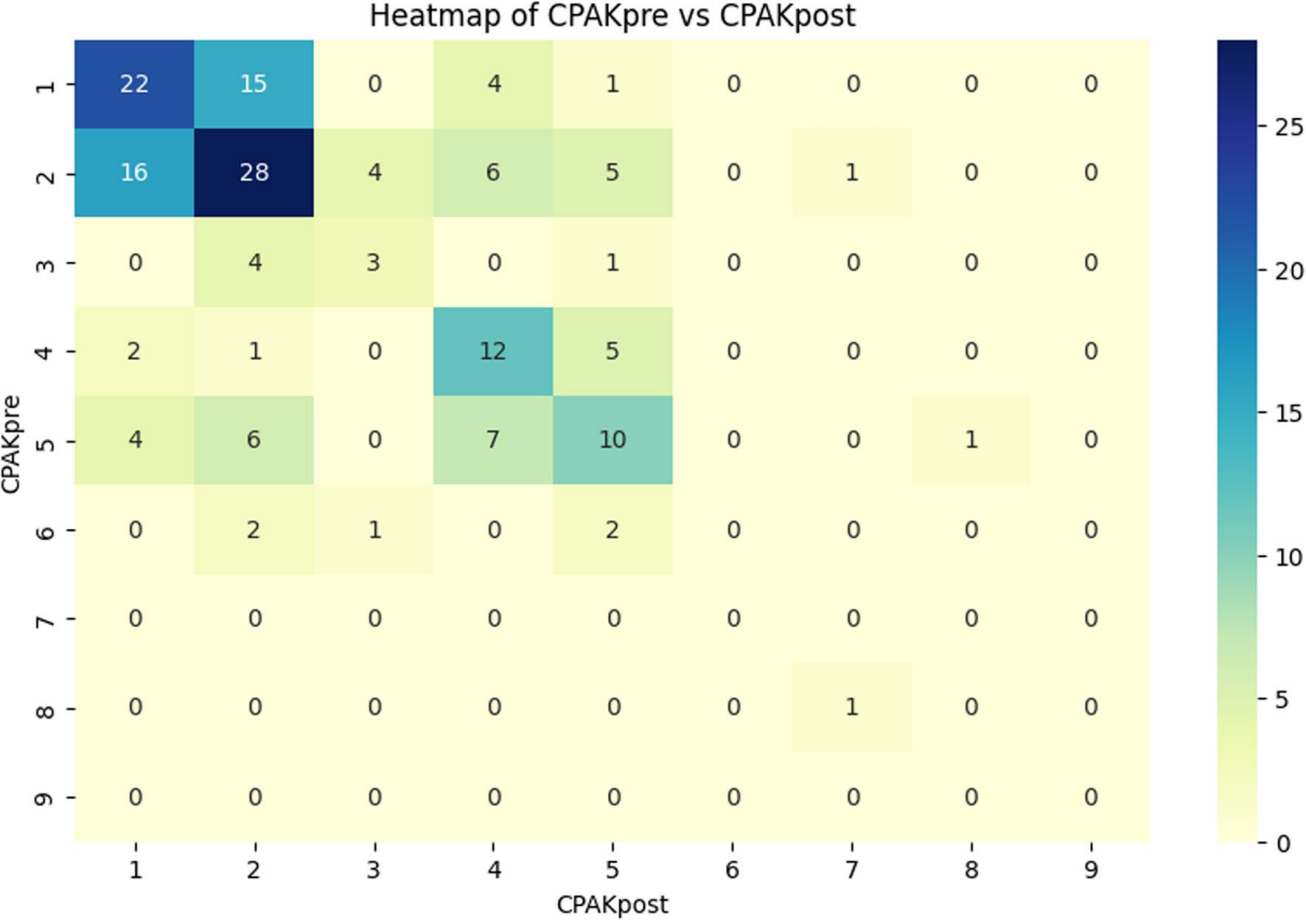
Heat map illustrating the transition of CPAK types from preoperative to postoperative. The *y* axis represents preoperative CPAK types, while the *x* axis represents postoperative CPAK types. Each cell in the heat map corresponds to the number of patients who transitioned from a specific preoperative CPAK type to a specific postoperative CPAK type. The darkness of the cell indicates the number of patients in each category, with darker cells representing a higher number of patients. CPAK, Coronal plane alignment of the knee; CPAKpre, preoperative CPAK; CPAKpost, postoperative CPAK

The presentation of preoperative and postoperative patient distribution of the CPAK types is depicted in Fig. 2. Among the patients who underwent UKA, 75 maintained their original CPAK type, whereas 89 experienced a change in the CPAK type. The transition of the CPAK type from preoperative to postoperative status is visualized through a heat map (Fig. 3).

The preoperative MPTA, LDFA, and HKA in patients who retained their CPAK type were 86.8 ± 1.9, 88.7 ± 2.1, and −6.0 ± 3.0 degrees, respectively. In contrast, those who experienced changes in their CPAK type had preoperative measures of 87.4 ± 1.9, 88.6 ± 1.6, and −6.2 ± 3.3 degrees, respectively. Comparisons of preoperative MPTA, LDFA, and HKA between the two groups showed no significant differences (*p* = 0.080, *p* = 0.627, *p* = 0.619, respectively).

A comparison of PROMs between patients who retained their original CPAK type and those who experienced alterations in the CPAK type is presented in Table 3. No significant differences were observed in preoperative PROMs between the two groups. However, postoperative PROMs, including the 1 year HSS score, KS knee score, and WOMAC pain score, were significantly superior in patients who did not experience any CPAK changes (*p* = 0.042, *p* = 0.009, and *p* = 0.048, respectively). Other scores also exhibited a tendency to favor patients who did not experience any CPAK changes, although these differences were not statistically significant. When subcategorizing the patients based on CPAK changes, 73 were classified into the minor CPAK change group, while 16 patients were categorized into the major CPAK change group. A statistically significant difference was observed in the 1 year KS knee score (*p* = 0.019), indicating superior outcomes in patients who did not experience any CPAK changes compared with those in the minor and major CPAK change groups (Table 4). Meanwhile, no significant difference was found between the minor and major CPAK change groups.

**Table 3:** Comparison of patient-reported outcome measures between patients with retained and altered CPAK types

	No CPAK change (*n =* 75)	CPAK change (*n =* 89)	*p*-Value
Preoperative HSS score	63.0 ± 12.8	62.2 ± 17.1	0.790
Preoperative KS knee score	53.2 ± 18.1	54.8 ± 15.8	0.669
Preoperative KS function score	38.0 ± 16.5	38.0 ± 16.3	1.000
Preoperative WOMAC pain	10.7 ± 3.8	10.7 ± 4.7	0.939
Preoperative WOMAC stiffness	4.1 ± 1.9	3.9 ± 2.0	0.755
Preoperative WOMAC physical function	34.5 ± 7.4	32.1 ± 8.3	0.152
Preoperative WOMAC total	49.2 ± 10.3	46.8 ± 10.7	0.275
Preoperative FJS	9.8 ± 9.5	12.3 ± 11.6	0.606
1Y HSS score	88.1 ± 9.5	83.3 ± 13.2	0.042*
1Y KS knee score	90.4 ± 7.1	86.1 ± 10.9	0.009*
1Y KS function score	66.1 ± 18.9	63.0 ± 19.9	0.384
1Y WOMAC pain	2.9 ± 3.0	4.1 ± 3.9	0.048*
1Y WOMAC stiffness	1.6 ± 1.6	2.0 ± 1.8	0.208
1Y WOMAC physical function	15.4 ± 8.9	16.8 ± 10.8	0.422
1Y WOMAC total	19.8 ± 11.9	22.9 ± 14.7	0.204
1Y FJS	56.3 ± 25.1	56.9 ± 20.6	0.929

CPAK, Coronal plane alignment of the knee; HSS, the Hospital for Special Surgery; KS, the Knee Society; WOMAC, the Western Ontario and McMaster Universities Arthritis Index; FJS, Forgotten Joint Score

*Statistically significant at *p* < 0.05

**Table 4:** Comparison of patient-reported outcome measures based on the degree of CPAK change

	No CPAK change (*n* = 75)	Minor CPAK Change (*n* = 73)	Major CPAK Change (*n* = 16)	*p*-Value
Preoperative HSS score	63.0 ± 12.8	63.4 ± 17.2	52.0 ± 14.0	0.281
Preoperative KS knee score	53.2 ± 18.1	56.9 ± 15.0	41.5 ± 15.4	0.104
Preoperative KS function score	38.0 ± 16.5	38.6 ± 16.9	34.7 ± 12.6	0.850
Preoperative WOMAC pain	10.7 ± 3.8	10.8 ± 4.8	10.6 ± 3.7	0.991
Preoperative WOMAC stiffness	4.1 ± 1.9	3.8 ± 2.2	4.1 ± 0.7	0.904
Preoperative WOMAC physical function	34.5 ± 7.4	31.3 ± 8.3	37.0 ± 7.0	0.075
Preoperative WOMAC total	49.2 ± 10.3	46.0 ± 10.7	51.7 ± 9.6	0.225
Preoperative FJS	9.8 ± 9.5	10.1 ± 12.1	17.7 ± 10.3	0.644
1Y HSS score	88.1 ± 9.5	82.8 ± 13.7	86.0 ± 9.8	0.130
1Y KS knee score	90.4 ± 7.1	86.8 ± 10.1	82.4 ± 14.5	0.019*
1Y KS function score	66.1 ± 18.9	63.1 ± 19.8	62.6 ± 21.0	0.688
1Y WOMAC pain	2.9 ± 3.0	4.1 ± 3.8	4.5 ± 4.3	0.155
1Y WOMAC stiffness	1.6 ± 1.6	2.0 ± 1.7	1.7 ± 2.2	0.409
1Y WOMAC physical function	15.4 ± 8.9	16.7 ± 10.6	17.2 ± 12.3	0.732
1Y WOMAC total	19.8 ± 11.9	22.8 ± 14.3	23.4 ± 17.6	0.467
1Y FJS	53.3 ± 25.1	58.3 ± 19.7	50.0 ± 26.0	0.754

CPAK, Coronal plane alignment of the knee; HSS, the Hospital for Special Surgery; KS, the Knee Society; WOMAC, the Western Ontario and McMaster Universities Arthritis Index; FJS, Forgotten Joint Score

*Statistically significant at *p* < 0.05

Throughout the study period, four patients required revision surgery. Among them, two underwent conversion to total knee arthroplasty due to tibial component loosening, which occurred at 82 months and 6 months postoperatively. Another patient required conversion to total knee arthroplasty due to a periprosthetic fracture of the proximal tibia resulting from a fall. Additionally, one patient developed periprosthetic infection 2 months after surgery and subsequently underwent arthroscopic debridement, without infection recurrence.

## Discussion

The main findings of this study revealed a significant difference in postoperative 1 year PROMs among patients who underwent UKA, and superior outcomes were observed in patients who did not experience any change in the CPAK types. The degree of CPAK change, whether minor or major, did not show any significant difference or trend in PROMs.

In this study, we utilized the CPAK classification as a novel approach to determine the optimal alignment following UKA. To the best of the authors’ knowledge, this is the first investigation to assess PROMs based on CPAK changes following UKA. Notably, patients who did not experience any CPAK changes demonstrated significant improvements in the HSS scores, KS knee scores, and WOMAC pain scores. In addition, an overall favorable trend was observed in the PROMs in these patients, with the exception of the FJS. This may suggest that while patients are likely to experience a significant improvement of PROMs postoperatively, those with preserved CPAK types likely experience greater pain relief and improved functional outcomes. Likewise, deviation from the native alignment may result in relatively less improvement in discomfort, pain, and decreased function compared with maintaining the original alignment. These findings, in the clinical setting, may encourage the surgeons to prioritize the patient’s native alignment when performing UKA.

Another strength of this study is the detailed categorization of CPAK changes into unchanged, minor, and major change groups. Interestingly, the findings revealed that the degree of change in the CPAK did not influence PROMs, compared with the change in CPAK itself. The number of patients with major CPAK changes was less (16 patients) than that of patients with minor CPAK changes (73 patients). This difference may be attributed to the fact that the UKAs assessed in this study were not aimed at overcorrecting the coronal alignment.

The optimal alignment target for UKA has been extensively studied; however, it has yielded inconclusive results. Alignment targets can be classified into the following three categories: overcorrection (including valgus alignment), undercorrection, and alignment resembling the patient’s native state. Studies supporting neutral or overcorrected alignment, such as those by Collier et al. and Whiteside et al., associated postoperative valgus alignment with a reduced risk of revision, despite potential unfavorable PROMs with extreme valgus knee angulation [7, 13]. Bayoumi et al. found that relative overcorrection from pre-arthritic alignment yielded improved mid-term outcomes compared with relative undercorrection [25]. In contrast, several studies have reported against valgus overcorrection in medial UKA [26–28]. Hernigou et al. demonstrated that valgus overcorrection of the coronal alignment increased the risk of cartilage degeneration in the lateral knee compartment [27]. Price et al. found that lateral knee compartment arthritis was the primary cause of revision in medial UKAs [29]. Additionally, a simulation study by Sekiguchi et al. suggested that a 2° varus to neutral alignment in the coronal plane was preferable compared with varus exceeding 4° or valgus alignment [9].

The postoperative changes in CPAK following medial UKA can primarily be attributed to changes in the medial joint line. If the joint line remains unchanged, it is expected that CPAK would also remain consistent. However, several factors can lead to variations in the joint line. In cases where tibial resection is minimal, and the gap is adjusted through additional femoral resection, the medial joint line may elevate, resulting in changes in joint line obliquity from apex distal to either neutral or apex proximal. Conversely, excessive tibial resection may have the opposite effect. Additionally, correction of flexion contracture through soft tissue release or bone resection aimed at achieving an appropriate extension gap may also contribute to change in CPAK.

There are several limitations to this study. First, its retrospective nature and the relatively small sample size increased the risk for introducing selection bias. In addition, 1 year PROMs were used as the primary outcomes, and longer follow-up periods are needed to assess any postoperative complications, including implant longevity. Third, the absence of a standardized approach for measuring the postoperative MPTA and LDFA in patients who underwent UKA necessitated designation of the center of the lateral joint space as a landmark. Although this method can provide a foundation for future investigations, its application should be interpreted cautiously.

## Conclusion

Preserving the native CPAK in UKA procedures is important for achieving favorable clinical outcomes at 1 year postoperative. The extent of change in the CPAK type exerted a limited impact on PROMs; thus, emphasizing the importance of change in alignment itself.

## Data Availability

The datasets used and/or analyzed during the current study are available from the corresponding author on reasonable request.

## Abbreviations

aHKA: Arithmetic hip–knee–ankle angle
CPAK: Coronal plane alignment of the knee
JLO: Joint line obliquity
LDFA: Lateral distal femoral angle
MPTA: Medial proximal tibial angle
PROM: Patient-reported outcome measures
UKA: Unicompartmental knee arthroplasty
